# Estimating the Allele-Specific Expression of SNVs From 10× Genomics Single-Cell RNA-Sequencing Data

**DOI:** 10.3390/genes11030240

**Published:** 2020-02-25

**Authors:** Prashant N. M., Hongyu Liu, Pavlos Bousounis, Liam Spurr, Nawaf Alomran, Helen Ibeawuchi, Justin Sein, Dacian Reece-Stremtan, Anelia Horvath

**Affiliations:** 1 McCormick Genomics and Proteomics Center, School of Medicine and Health Sciences, The George Washington University, Washington, DC 20037, USA; pnm27@gwmail.gwu.edu (P.N.M.); hliu5259@gwu.edu (H.L.); pdbous@gwu.edu (P.B.); lspurr@broadinstitute.org (L.S.); naa71@georgetown.edu (N.A.); hibeawuchi@gwmail.gwu.edu (H.I.); jsein@gwu.edu (J.S.); 2 Chinese Medicine Toxicological Laboratory, Institute of Traditional Chinese Medicine, Heilongjiang University of Chinese Medicine, Harbin 150040, China; 3 Department of Medical Oncology, Dana-Farber Cancer Institute, Boston, MA 02215, USA; 4 Cancer Program, The Broad Institute of MIT and Harvard, Cambridge, MA 02142, USA; 5 Computer Applications Support Services, School of Medicine and Health Sciences, The George Washington University, Washington, DC 20037, USA; stremtan@gwu.edu; 6 Department of Biochemistry and Molecular Medicine, Department of Biostatistics and Bioinformatics, School of Medicine and Health Sciences, George Washington University, Washington, DC 20037, USA; 7 Department of Pharmacology and Physiology, School of Medicine and Health Sciences, The George Washington University, Washington, DC 20037, USA

**Keywords:** single cell, VAF_RNA_, sc-VAF_RNA_, sc-RNA-seq, monoallelic expression, SNV, genetic variation, RNA-seq, single-cell RNA-sequencing

## Abstract

With the recent advances in single-cell RNA-sequencing (scRNA-seq) technologies, the estimation of allele expression from single cells is becoming increasingly reliable. Allele expression is both quantitative and dynamic and is an essential component of the genomic interactome. Here, we systematically estimate the allele expression from heterozygous single nucleotide variant (SNV) loci using scRNA-seq data generated on the 10×Genomics Chromium platform. We analyzed 26,640 human adipose-derived mesenchymal stem cells (from three healthy donors), sequenced to an average of 150K sequencing reads per cell (more than 4 billion scRNA-seq reads in total). High-quality SNV calls assessed in our study contained approximately 15% exonic and >50% intronic loci. To analyze the allele expression, we estimated the expressed variant allele fraction (VAF_RNA_) from SNV-aware alignments and analyzed its variance and distribution (mono- and bi-allelic) at different minimum sequencing read thresholds. Our analysis shows that when assessing positions covered by a minimum of three unique sequencing reads, over 50% of the heterozygous SNVs show bi-allelic expression, while at a threshold of 10 reads, nearly 90% of the SNVs are bi-allelic. In addition, our analysis demonstrates the feasibility of scVAF_RNA_ estimation from current scRNA-seq datasets and shows that the 3′-based library generation protocol of 10×Genomics scRNA-seq data can be informative in SNV-based studies, including analyses of transcriptional kinetics.

## 1. Introduction

In the last several years, single-cell RNA-sequencing (scRNA-seq) has become an accessible platform for genomic studies [1,2,3]. By enabling cell-level transcriptome analyses, scRNA-seq exhibits a major advantage over the conventional averaged bulk RNA-seq, which is the ability to assess intracellular relationships between molecular features. With the emerging advances in scRNA-seq technologies, estimations of genetic variation from scRNA-seq data are becoming more reliable [4,5,6]. Recent studies have demonstrated the usefulness of scRNA-seq single nucleotide variant (SNV) assessments for a variety of applications, including random monoallelic expression (RME), transcriptional burst kinetics [7,8,9,10,11], haplotype inference [12], chromosome X inactivation [13,14], genetic heterogeneity in cancer [15,16,17,18,19], aneuploidy [20], quantitative trait loci (QTL) assessments [21], and demultiplexing [22,23,24].

Genetic variants are traditionally called from DNA and often analyzed and interpreted as discrete genotypes (for diploid organisms, homo- or heterozygous). For expressed loci, genetic variation can also be assessed using RNA-seq data [24,25,26,27,28,29,30], by calculating the variant allele fraction (VAF_RNA_ = n_var_/(n_var_ + n_ref_), where n_var_ and n_ref_ are the variant and reference read counts, respectively). VAF_RNA_ is an informative measure of genetic variation for several reasons. First, compared to the categorical genotypes (DNA allele count of 0, 1, and 2), VAF_RNA_ is a continuous measure allowing for precise allele quantitation, which is important for sites where VAF_RNA_ does not scale with the DNA allele count. These include loci exhibiting a preferential expression of functional alleles, somatic mutations in cancer, and RNA-editing loci. Second, in contrast to (static) genotypes, VAF_RNA_ is dynamic and reflects the actual allele content in the system at a specific moment in time, which aids the assessment of dynamic and progressive processes. Importantly, through primarily reflecting genetic variation, VAF_RNA_ is an essential component of the genomic interactome and plays a major role in phenotype formation [31,32,33,34,35].

However, a systematic analysis of the feasibility of VAF_RNA_ estimations from 3′-based scRNA-seq libraries and its usefulness for addressing biological questions has not yet been performed. One of the basic biological processes assessed through VAF_RNA_ is the prevalence of RME across the diploid mammalian genome. Several recent scRNA-seq studies have described widespread RME in both human and murine models [7,8,9,10,11]. Most of these studies analyzed scRNA-seq data generated on full-length transcript platforms from hundreds of cells.

Here, we demonstrate a pipeline to estimate VAF_RNA_ from scRNA-seq data obtained from the 10×Genomics Chromium platform [36]. We selected this platform due to its growing popularity, along with its (1) high throughput (our analysis includes 26,640 cells obtained from three healthy donors), (2) support for unique molecular identifiers (UMI) for the removal of PCR-related sequencing bias, and (3) high sequencing depth compared to other 10×Genomics datasets (~150,000 sequencing reads per cell). Because VAF_RNA_ is sensitive to allele-mapping bias, we used SNV-aware alignments where reads mapped ambiguously due to the variant nucleotide(s) are being removed [37]. To assess the effects of the sequencing depth on the VAF_RNA_ estimations, we used minR, which we defined as the number of unique sequencing reads required for the SNV locus to qualify for VAF_RNA_ estimation (i.e., positions covered by fewer reads than minR were not included in the analysis). From the SNV-aware alignments, we systematically assessed the ability to estimate VAF_RNA_ using three different minR cutoffs: minR = 3, minR = 5, and minR = 10. We compared outputs across thresholds and individuals, and outlined lists of consistent observations. We also demonstrate an approach for assessing RME, and compare the results from scRNA-seq data generated on the 10×Genomics Chromium with studies based on different platforms.

## 2. Materials and Methods 

### 2.1. Data

We used publicly available scRNA-seq data from 26,640 human adipose-derived stem cells (ADSC) from three healthy donors (N8, N7, and N5); the scRNA-seq data was generated on the 10×Genomics Chromium v2 platform [36]. The library preparation and sequencing are described in detail elsewhere [36]. Briefly, cells were partitioned using 10× Genomics Single Cell 3′ Chips, and barcodes to index cells (14 bp), as well as transcripts (10 bp UMI), were incorporated. The constructed libraries were sequenced on an Illumina NovaSeq 6000 System in a 2 × 150 nucleotides (nt) paired-end mode.

### 2.2. scRNA-seq Data Processing

**Figure 1:**
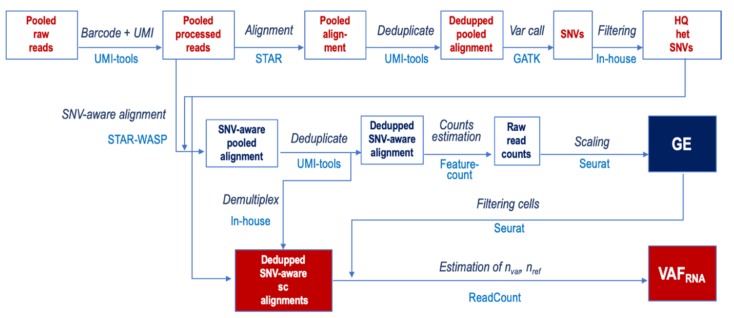
Analytical workflow for an estimation of the variant allele fraction VAF_RNA_ from single-cell RNA-sequencing (sc-RNA-seq) data.

The processing pipeline is shown in Figure 1. First, we extracted the cell barcodes and UMIs using UMI-tools from the pooled (per donor) raw sequencing reads [38].

**Figure 2:**
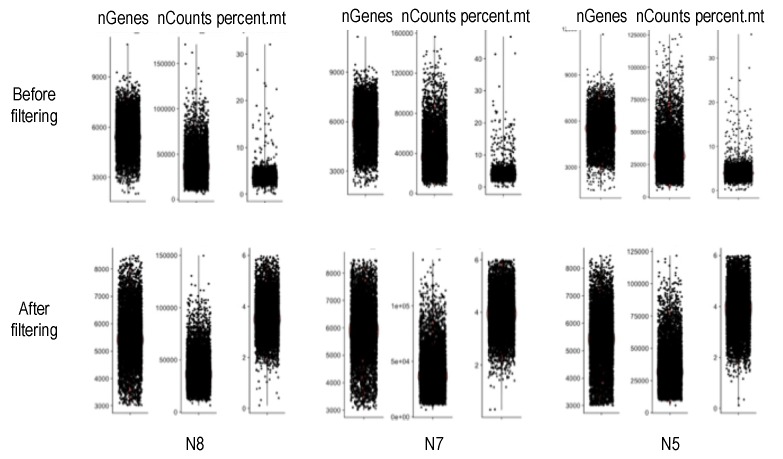
Number of genes, number of sequencing reads, and percent of mitochondrial genes for N8, N7, and N5 before (top) and after (bottom) the filtering out of cells with low-quality data.

Next, we aligned the reads to the latest version of the human genome reference (GRCh38, Dec 2013) using STAR v.2.7.3.c [39] in 2-pass mode with transcript annotations from the assembly GRCh38.79. After deduplication with UMI-tools, we called SNVs in the pooled alignments using GATK v.4.1.4.1 [26]. Heterozygous SNVs were selected based on the presence of at least 50 high-quality reads supporting both (reference and alternative) nucleotides in the pooled alignments. From those, we retained heterozygous SNVs meeting the following requirements for further analysis: QUAL (Phred-scaled probability) > 100, MQ (mapping quality) > 60, QD (quality by depth) > 2, and FS (Fisher’s exact test estimated strand bias) = 0.000. In addition, we used annotation (SeattleSeq v.13.00, dbSNP (The Single Nucleotide Polymorphism database) build 153) to remove SNV loci positioned in repetitive or intergenic regions. The SNV lists for each donor were then used as an input for a second, SNV-aware alignment using STAR, this time including the WASP-option [37,39] for the removal of reads mapped ambiguously due to the variant nucleotide. The SNV-aware alignments were deduplicated, keeping the reads with the highest mapping scores using the UMIs, and demultiplexed using the cell barcodes. Raw gene counts were estimated using featureCount [40], after which they were normalized and scaled using Seurat v.3.0 [41]. These gene counts were then employed to remove cells with low-quality data, defined as <3000 detected genes or a mitochondrial gene expression higher than 6% of the total gene expression. The before- and after-filtering distributions of genes and RNA-seq reads are shown in Figure 2.

We estimated VAF_RNA_ from the individual alignments of cells with high-quality data using ReadCounts [31]: VAF_RNA_ = n_var_/(n_var_ + n_ref_), where n_var_ and n_ref_ are the variant and reference read counts, respectively. Next, we performed analyses of VAF_RNA_ estimations obtained at three different cutoffs for the required number of reads (minR): minR = 10, minR = 5, and minR = 3. For each analysis, minR was kept constant across the genome, and positions covered by fewer reads than minR were not included in the analysis.

## 3. Results

### 3.1. Overall Findings

The numbers of individual single cells with high-quality data retained for further analysis were 9115, 8125, and 8533 for N8, N7, and N5, respectively. From these cells, we estimated VAF_RNA_ in 50,532 SNV genomic positions in N8, 61,407 in N7, and 38,822 in N5, which were the number of genomic positions retained after filtering for heterozygosity, the quality of the cell, and the position in intragenic non-repetitive regions. To support multi-cell estimations, we only retained positions for which VAF_RNA_ was estimated in a minimum of 10 individual cells for statistical analysis. Accordingly, unless otherwise indicated, the hereafter presented analyses are assessments from a minimum of 10 cells (per donor). For minR = 10, the absolute number of these positions was 366, 431, and 277 for N8, N7, and N5, respectively. This number was approximately 4-fold higher for positions assessed at minR = 5 and up to 20-fold higher for positions at minR = 3; the outputs are summarized in Table 1. We note that the relaxed thresholds are inclusive of the more stringent ones (i.e., minR = 5 loci include the loci at minR = 10, etc.). Of note, between 6% and 14% of all captured SNVs have been previously associated with a clinical phenotype or highlighted by genome-wide association studies (GWAS) analyses (See Table 1).

Overall, our analysis shows that the analyzed scRNA-seq datasets generated on the 10× Genomics platform contain a considerable number of expressed SNVs covered by at least three unique sequencing reads. At all depth thresholds, approximately 10% of the SNVs have been associated with (or assessed for an association with) a phenotype, which indicates that 10× Genomics data can be used in studies examining the functionality of genetic variants.

### 3.2. Position-Based SNVs Annotation

**Figure 3:**
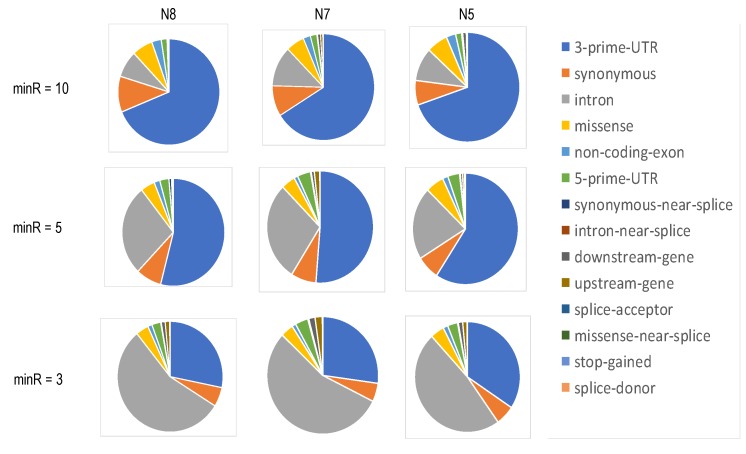
Functional annotation (based on the position in regard to the harboring genes) of SNVs captured by the 10×Genomics platform with different required minimal counts of unique sequencing reads. At minR = 5, over 45% of the SNVs are positioned downstream of the 3′-UTR regions.

To assess the distribution of SNVs in regard to their position in the gene and predicted functionality, we annotated the SNVs via SeattleSeq (v13, dbSNP build 153); the distribution of functional annotations at each of the three thresholds is shown in Figure 3.

At minR = 10, close to three-quarters of the captured SNVs were positioned in the 3′-UTRs of the transcripts, while at minR = 5, this proportion decreased to slightly over 50%. At minR = 3, approximately a quarter of the captured SNVs resided in the 3′UTR, while the intronic SNVs increased in proportion to more than 50%. At all thresholds, over 15% of the SNVs were exonic. The complete annotations are shown in Supplementary Tables S1–S3.

Our position-based SNV analysis shows that, as expected from a 3′-based platform, the largest proportion of called SNVs reside in the 3′UTR. Of note, this percentage varies inversely with minR. If we exclude technical factors (including erroneous priming during the library preparation), this observation may indicate a low-level expression of transcripts with alternative polyadenylation sites. Importantly, we also observed a substantial percentage of SNVs located in non-3′UTR gene regions (including exons), which indicates that 10× Genomics scRNA-seq data can be used for an analysis of SNVs from different functional categories.

### 3.3. Allele Expression from Single Cells at an SNV Level

To assess the allele expression from single cells, we analyzed all SNV loci covered with the required number of sequencing reads (minR = 10, 5, and 3) in at least 10 individual cells. For each SNV locus, we computed a number of VAF_RNA_ statistics, including the mean, median, and percentage of mono- and bi-allelic expressing cells (see also Supplementary Tables S1–S3). At all thresholds, the distributions of the VAF_RNA_ mean and median values were generally symmetrical in regard to the VAF_RNA_ scale (Supplementary Figure S1). Additionally, at all thresholds, more than half of the VAF estimations were in the range of 0.2 < VAF_RNA_ < 0.8, corresponding to bi-allelic expression (Table 2). Specifically, VAF_RNA_ obtained at minR = 3 corresponded to bi-allelic expression for over 50% of the estimations, and this proportion increased to approximately 90% when confining the analysis to VAF_RNA_ estimated at minR = 10.

**Figure 4:**
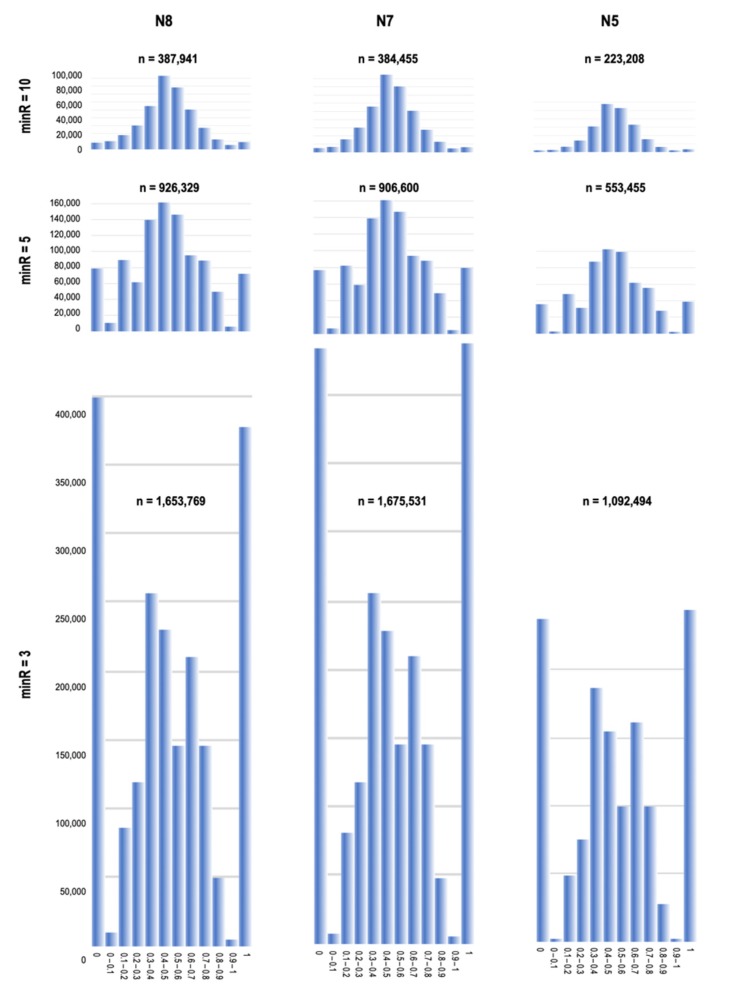
Histograms representing the distribution of scVAF_RNA_ at minR = 10 (top), minR = 5 (middle), and minR = 3 (bottom) for all the heterozygous SNVs in N8, N7, and N5. The bin width (x-axis) is 0.1; bin intervals are indicated in the middle of each plot. The y-axes show the numbers of VAF_RNA_ measurements in the individual cells. The total number of VAF_RNA_ estimations (n, across all the cells per group) is shown at the top of each histogram. The histograms are scaled in regard to the number of cells. Across the entire dataset, at minR = 10 and minR = 5, the majority of SNVs showed bi-allelic expression centered around a VAF_RNA_ value of 0.5 (0.4 < VAF_RNA_ < 0.6). In contrast, at minR = 3, the majority of SNVs presented with strict monoallelic expression (VAF_RNA_ = 0 or 1). The VAF_RNA_ distributions showed remarkable similarity across the three individuals (N8, N7, and N5).

The distribution of scVAF_RNA_ estimations at minR = 10, 5, and 3 for all heterozygous SNVs in corresponding samples is shown in Figure 4; the histograms include bins for strictly monoallelic expression, defined as VAF_RNA_ values of 0 and 1. Markedly, the data obtained using different minR cutoffs resulted in different scVAF_RNA_ distributions. At minR = 3, the VAF_RNA_ distribution showed a considerable proportion of calls corresponding to monoallelic expression. These monoallelic calls may result from both the stochasticity of sampling, which impacts positions covered by a few reads, and RME, reported previously in single-cell studies [7,8,9,10,11]. At minR = 5, strictly monoallelic VAF_RNA_ estimations represented less than half of those with VAF_RNA_ = 0.5 + 0.1, and decreased in proportion to below 5% at minR = 10.

**Figure 5:**
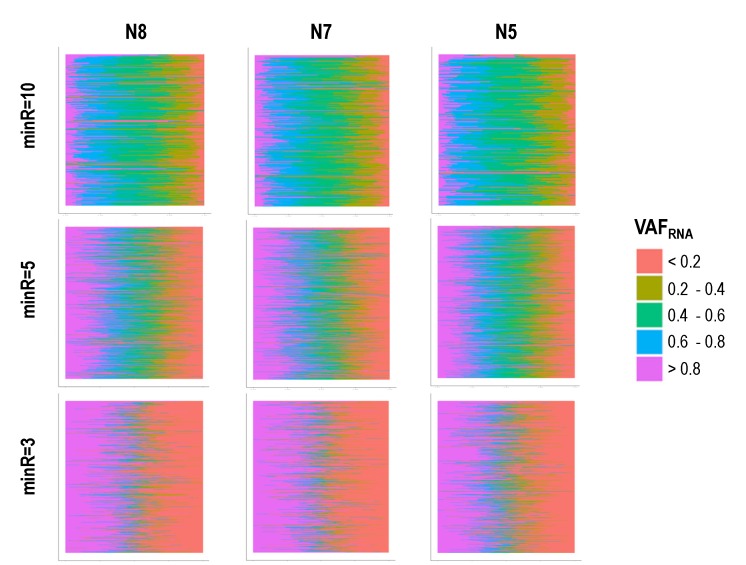
scVAF_RNA_ estimated at positions covered by a minimum of 10 sequencing reads (top), 5 sequencing reads (middle), and 3 sequencing reads (bottom), across more than 1000 cells. For the majority of positions, VAF_RNA_ showed bi-allelic expression, with a substantial proportion of the scVAF_RNA_ estimations in the interval 0.4–0.6.

Next, we analyzed the data per SNV; VAF_RNA_ distributions for all the SNVs (genome-wide) assessed from a minimum of 1000 cells are plotted in Figure 5 and Supplementary Figure S2. Aligned with the above observations, at minR = 10, the majority of SNV positions had a substantial proportion of cells with VAF_RNA_ estimations between 0.2 and 0.8; this proportion gradually decreased at lower thresholds.

Overall, our results show that the VAF_RNA_ distribution depends on the minimum number of reads required for an SNV to qualify for inclusion in the analysis. When we include only positions covered by a high number of reads (i.e., 10), the vast majority of VAF_RNA_ estimations suggest bi-allelic expression; these analyses are confined to a relatively low number of SNVs (i.e., hundreds of SNVs per sample estimated in at least 10 cells). Lowering the minR naturally results in a higher number of SNVs; in this larger group, we observe higher rates of monoallelic calls. Because at low minR stochasticity of sampling can affect the VAF_RNA_ estimations, the observed high rate of monoallelic VAF_RNA_ calls at minR = 3 could be a result of both technical and biological factors. To assess the effects of technical factors on our analysis, we estimated the consistency of the VAF_RNA_ measurements from multiple SNV loci of the same gene, and across the three different samples; we also compared the findings with those from previous scRNA-seq studies (Section 3.4 below).

### 3.4. Allelic Expression from Single Cells at a Gene Level

We analyzed VAF_RNA_ at a gene level and compared the findings to those from a major study on allele-specific expression from human scRNA-seq data (Borel *et al* [9]), using similar definitions for allele-specific expression. Specifically, as monoallelic expression (including RME), we defined SNVs for which fewer than 5% of the cells displayed a VAF_RNA_ value between 0.2 and 0.8 (0.2 < VAF_RNA_ < 0.8). Skewed allelic expression was assigned to SNVs where less than 10% of the cells expressed one type of allele and the rest expressed either the second allele or both alleles (<80% cells with 0.2 < VAF_RNA_ < 0.8).

**Figure 6:**
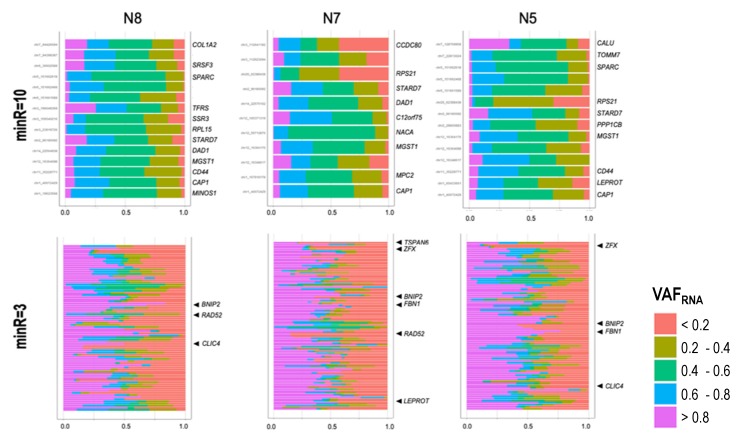
scVAF_RNA_ distribution at positions covered by a minimum of 10 sequencing reads (top), and three sequencing reads (bottom), across more than 1500 cells for genes reported by Borel et al. [9]. For the positions with minR = 10, no RME was suggested by the scVAF_RNA_ distribution for autosomal genes (i.e., for the majority of the estimations scVAF_RNA_ values were between 0.2 and 0.8), while positions covered with minR = 3 showed frequent monoallelic signals (scVAF_RNA_ > 0.8 or scVAF_RNA_ < 0.2). As expected, chrX shows strong RME patterns (see gene *TSPAN6*).

We analyzed the genes common to both the dataset used in Borel et al. [9] and our dataset; for our dataset this analysis was performed at all three thresholds. We first assessed the findings at minR = 10, where 21 genes from our dataset were present among the 60 genes highlighted in Borel et al. [9] (Figure 6, top); all 21 genes showed consistent bi-allelic expression in the two studies. From the above-mentioned 60 genes, autosomal genes with RME were only observed at minR = 3 in our dataset, all of which were in complete concordance with Borel et al. [9]. Examples of such genes are shown (Figure 6, bottom), including the strictly monoallelic *RAD52*. Out of the 12 genes with a reported skewed allelic expression, four were present in our dataset: *CNN3*, *C12orf75*, and *CCDC80* had a skewed expression, while *SPC3* showed symmetrically distributed alleles in both samples where it was detected (Supplementary Table S3).

**Figure 7:**
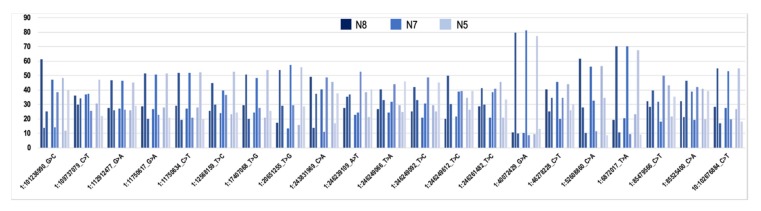
Percentage of cells (y-axis) displaying VAF_RNA_ < 0.2 (for each cluster of three, left), VAF_RNA_ between 0.2 and 0.8 (middle), and VAF_RNA_ > 0.8 (right); minR = 10. High concordance between the three donors is seen; SNVs on chromosome 1 are shown, and the results were similar genome-wide.

We next analyzed the concordance of VAF_RNA_ estimations between multiple SNVs residing in the same gene. Markedly, at minR = 10, we observed concordant allelic expression for all genes with more than one SNV (see *COL1A2*, *SPARC*, *CCDC80*, and *MGST1* in Figure 6). We also observed complete concordance across the three individuals for the SNVs shared between donors; SNVs common for the three donors and assessed from more than 50 cells per donor are shown in Figure 7 (chromosome 1, the rest of the chromosomes showed similar results; see also *CAP1*, *DAD1*, *SPARC*, *MGST1*, *CD44*, and *STARD7* in Figure 6).

Next, we assessed mono- and bi-allelic SNV-expression at a gene level across our entire dataset at minR = 3. We confined this assessment to SNVs seen in a minimum of 50 cells per sample; 7408 SNVs in 3406 genes were eligible for this analysis across the three donors. Predominant RME (fewer than 5% of the cells with VAF_RNA_ between 0.2 and 0.8) was seen in 451 SNVs positioned in 376 genes; from those, 49 SNVs in 42 genes did not have any cells expressing both alleles.

**Figure 8:**
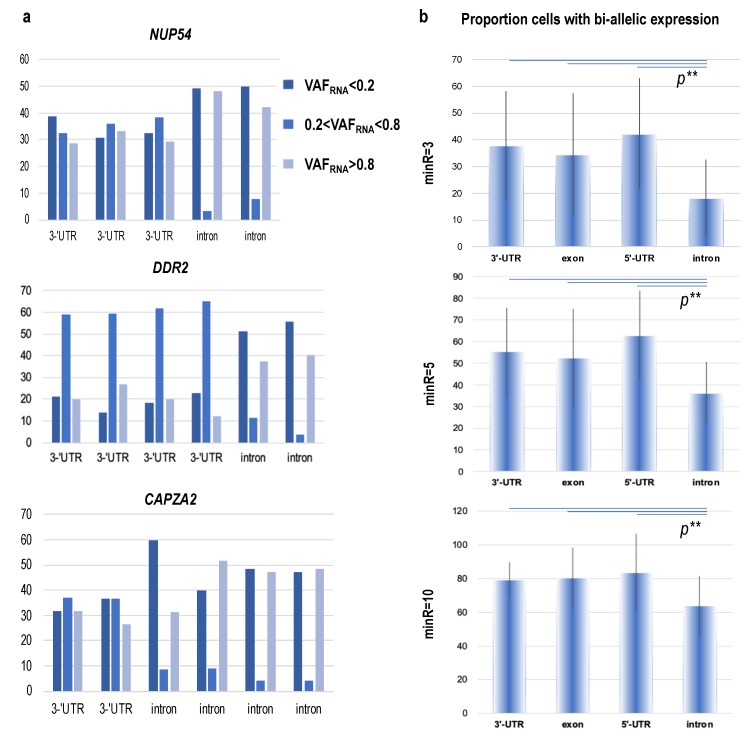
(**a**) Genes with multiple SNVs positioned in intronic and non-intronic sequences; high percentage of random monoallelic expression (RME) cells (VAF_RNA_ < 0.2 or > 0.8, y-axis) is obvious. (**b**) Average percentage of cells (y-axis) with bi-allelic expression across all SNVs in our dataset stratified by position in the gene; SNVs positioned in introns were be-allelic in a lower proportion of cells as compared to all other SNVs.

We next assessed the genome-wide consistency of the VAF_RNA_ across multiple SNVs from the same gene. To do this, we pooled the SNVs from the three donors together and selected genes with more than three SNVs, each assessed from a minimum of 50 cells per donor; 3922 SNVs in 815 genes were available for this analysis. The first striking observation was that, for most of the genes, intronic SNVs had substantially higher rates of monoallelic calls compared to SNVs in the spliced mRNA (Figure 8). This was evident both at the level of the individual genes (examples shown in Figure 8a), and genome-wide, where the average proportion of cells expressing both alleles (0.2 < VAF_RNA_ < 0.8) was significantly lower for SNVs positioned in introns, as compared to SNVs in exons and UTRs (Figure 8b, chi-square test, *p* < 0.05 for all the comparisons at all three thresholds). Within the groups of intronic and non-intronic SNVs in the same gene, highly consistent VAF_RNA_ distributions were observed.

Our analysis shows highly consistent scRNA-seq VAF_RNA_ estimations from positions covered by a minimum of 10 unique sequencing reads. Furthermore, multiple SNVs from the same gene showed lower rates of bi-allelic expression from intronic (as compared to non-intronic) SNVs across the three thresholds. However, we note that the latter observation can be affected by the lower counts of intronic (as compared to spliced) RNAseq reads, where a stochasticity of sampling is expected to have a high impact, especially at minR = 3.

### 3.5. Considerations for VAF_RNA_ Estimations From 10× Genomics scRNA-seq Data 

We note several important considerations for VAF_RNA_ estimations from scRNA-seq data generated on the 10×Genomics Chromium Platform. First, as mentioned earlier, this scRNA-seq data is confined to 3′-targeted, relatively short (150nt in our study) sequencing reads. These reads cover only a proportion of the SNVs residing in a transcript, and, for many genes, are likely to not cover a large proportion of the SNVs. Therefore, this data is not suitable for full-length transcript SNV analyses.

Second, observations of monoallelic expression from analyses at low read-count cutoffs can result from both biological (i.e., RME) and technical factors (stochasticity of sampling). Specifically, at minR = 3, there is a considerable probability of erroneously assigning a monoallelic status to bi-allelic positions (false positive) due to stochastic factors. We assessed the confidence of the VAF_RNA_ estimations by checking for the consistency of observations at the following levels: (1) between multiple SNVs in the same gene, where we observed high concordance (see Figure 8a); (2) across different samples, where we also observed concordant VAF_RNA_ estimations (see Figure 7); and (3) with previous estimations [9]. Moreover, to define a VAF_RNA_ pattern for a certain SNV, we used information from a minimum of 50 individual cells. Our observation of high rates of monoallelic expression at minR = 3 is consistent with a major study on allele-specific expression from human and mouse scRNA-seq data (Deng et al, [10]), which found stable bi-allelic expression for only a few hundred genes, often with housekeeping functions. Furthermore, the authors reported the mean gene expression levels in cells with bi-allelic expression to be approximately two-fold higher than the levels in cells with monoallelic expression. Low read-count cutoffs are generally expected to include a higher number of low-expressed genes. For low-expressed genes, additional technical noise can affect the estimations; therefore, findings at low read cutoffs need to be considered with caution, and validated through additional analyses or experiments.

Related to the above, when selecting minR for an analysis, a major factor to be considered is the balance between the confidence of VAF_RNA_ estimation (high minR) and the number of analyzed SNVs (a lower minR will naturally qualify more SNV loci for VAF_RNA_ estimations). Our data shows that for current scRNA-seq datasets produced on the 10× Genomics platform, minR = 5 provides a reasonable balance between confidence of the VAF_RNA_ estimation and the number of SNVs. For higher confidence, we suggest analyzing the data with more than one minR in parallel, and assessing the concordance between the more inclusive results at a low minR and the more confident observations at a high minR. 

Furthermore, quality control (QC)-related factors can also affect the estimation of the VAF_RNA_ distribution. These include (1) incorrect variant calls (i.e., inaccurate assignment of the presence or absence of an SNV at a given genome position for which VAF_RNA_ is to be estimated), (2) an inaccurate assignment of the heterozygous SNV state, and (3) VAF_RNA_ estimation. Methods for SNV calls from scRNA-seq data are currently being optimized and benchmarked [4,5,6]. In this pilot study on the scVAF_RNA_ distribution, we only focused on highly confident SNV calls by retaining for analysis the SNVs (a) with the highest mapping and Phred call quality, (b) positioned outside repetitive regions (known to challenge SNV estimations), and (c) previously validated through dbSNP. In addition, we note that we called SNVs from the pooled (across all the cells per sample) alignments, which helps reduce challenges related to variant calling from scRNA-seq data, and to increase the confidence of heterozygous estimations. Furthermore, when estimating VAF (3), a major factor is the allele-specific mapping bias, which we corrected using WASP [37]. WASP is implemented in the latest versions of the herein used popular aligner STAR [26], which significantly streamlines data processing, especially for datasets with predefined lists of SNV loci of interest (i.e., available genotypes, lists of known SNVs of interest such as RNA-editing sites, dbSNP, etc.).

Finally, the presented pipeline uses RNA-seq data only. While our approach is designed for datasets where matched DNA is not available, one should note that in such a setting, assigning a heterozygosity status for certain SNVs (for example, the SNVs residing in imprinted genes) may be challenging. To confidently assign heterozygosity, we confined our study to bi-allelic SNVs, for which we required a minimum of 50 unique reads supporting each allele from the pooled RNA-seq data per donor. By default, this selection excludes heterozygous SNVs with strong non-random monoallelic expression (which would appear as monoallelic in the pooled RNA-seq data). Therefore, the herein presented results need to be considered strictly in the light of this selection. For datasets with available DNA, we recommend the use of genotype calls for assigning a heterozygosity status.

## 4. Discussion

Our analysis includes more than 4 billion RNA-seq reads and over 7.8 million individual scVAF_RNA_ estimations, making it, to our knowledge, the largest study on SNV-based allele-specific expression from human scRNA-seq data. We leveraged a large number of cells (over 24K) and a high sequencing depth (150K reads per cell) to explore the feasibility of scVAF_RNA_ estimations, and defined a set of scVAF_RNA_ characteristics. Our results show that an SNV assessment of scRNA-seq generated through the 3′-based 10×Genomics platform can be highly informative for several reasons.

First, annotation of the captured variants supports analyses on variant functionality. As expected, 10×Genomics scRNA-seq data contains a significant proportion of 3′-UTR variants, which are known to strongly affect both gene expression and splicing [42,43,44,45,46]. In addition, approximately 15% of the captured SNVs are exonic, and include missense, nonsense, and near-splice variants, many of which can potentially affect the protein structure and function (see Supplementary Tables S1–S3). Importantly, the platform captures a substantial number of intronic SNVs. Intronic sequences are reported in 15%–25% of the RNA-sequencing reads from both bulk and single-cell-based studies [47,48,49,50]. ScRNA-seq intronic sequences can be used to estimate the relative abundance of precursor and mature mRNA, thereby assessing the RNA velocity and dynamic cellular processes [47]. Consistent with a major recent study on RNA velocity [47] and models of transcriptional burst kinetics [7], we observed a higher monoallelic expression for intronic SNVs as compared to non-intronic SNVs for a given gene (see Figure 8). Specifically, it was established that at times of increased transcription, unspliced precursors are rapidly produced (often from one of the alleles), and conversely, the proportion of unspliced mRNAs is quickly reduced during periods of lower transcription. Therefore, at any given moment, a single cell is likely to contain more unspliced precursors produced from one of the alleles as compared to the longer-lived spliced mRNAs of the same gene, which are more likely to accumulate both alleles over time. Because the balance of unspliced and spliced mRNA abundance is predictive of the future state of the mature mRNA [47], scVAF_RNA_ analyses can be applied to assess dynamic cellular processes. However, for such analyses, it is important to consider the generally lower intronic read counts (as compared to non-intronic) and the related increased probability of erroneously assigning monoallelic calls at bi-allelic positions.

Second, to our knowledge, this is the first study to estimate allele expression from a minimum of 10 unique sequencing reads from scRNA-seq data. Our findings indicate that at such stringency, the majority of autosomal genes show largely symmetric bi-allelic expression. We provide this data (minR = 10, Supplementary Table S1), together with the estimations at minR = 5 and minR = 3 (Supplementary Tables S2 and S3), so that it can be used for analyses of allele-specific expression, both genome-wide and at the level of individual genes of interest. 

Third, we have presented a set of characteristics of VAF_RNA_ obtained from scRNA-seq data. Several factors facilitate the applicability of VAF_RNA_ to assess functional genetic variants (from both bulk and scRNA-seq data). As mentioned earlier, VAF_RNA_ allows for precise allele quantitation, which is particularly important for sites with allele-specific regulation, RNA-editing, and somatic mutations in cancer. Furthermore, VAF_RNA_ is dynamic and reflects the actual allele content in the cell at a particular moment in time. In scRNA studies, where the different cells are often in gradual states of progressive processes, VAF_RNA_ analyses can be adopted to study lineages and cellular dynamics. Finally, VAF_RNA_ can be used to study functional SNVs from sets where matched DNA (and, respectively, genotypes) data is not available [29,30]. Ultimately, these analyses apply to expressed SNVs and will not capture loci positioned in transcriptionally silent regions. The single-cell resolution of this approach brings further advantages. First, due to the preservation of intracellular relationships between molecular features, single-cell analyses facilitate the discovery of correlations between SNVs and other transcriptome features, such as gene expression or splicing. Finally, scRNA-seq projects typically utilize cells with (largely) identical genotypes (i.e., from the same system/individual), thus supplying context for the assessment of SNVs implicated in RNA-specific regulation.

## 5. Conclusions

In conclusion, we have presented a large SNV-focused study on allele expression from scRNA-seq data that addresses three major technical factors known to bias single-cell allelic studies: PCR-related bias, allele-mapping bias, and a low number of sequencing reads. To facilitate similar studies, we have described a step-by-step approach for confident scVAF_RNA_ estimations. Our study is largely consistent with existing knowledge, reports findings on previously unassessed genes and SNVs, and supplies datasets for further analyses. In addition, our analysis demonstrates the feasibility of scVAF_RNA_ estimation from current scRNA-seq datasets and shows that the 3′-based library generation protocol of 10×Genomics scRNA-seq data can be highly informative for SNV-based analyses. 

## Figures and Tables

**Table 1:** Summary statistics for the scRNA-seq data of the three ADSC samples. We estimated the number of single nucleotide variant (SNV) loci covered in at least 10 individual cells (per donor) with different thresholds for the minimum number of unique reads (minR), and from those, the number of SNVs associated with a phenotype (phenotype and clinical associations were extracted via SeattleSeq database annotation (v13, dbSNP build 153)).

Sample	N Cells	N Reads	Mean Reads/Cell	Median Genes/Cell	N Cells Post Filtering	N Het SNVs (min 10 cells)			N SNVs With Phenotype/Clinics		
						minR 10	minR 5	minR 3	minR 10	minR 5	minR 3
**N8**	9256	1,285,218,728	138,852	5559	9115	366	1,567	7253	47	181	552
**N7**	8478	1,579,342,505	186,287	6049	8125	431	1,994	9032	57	184	568
**N5**	8906	1,071,156,174	120,273	5439	8533	277	1,134	5357	23	134	422

**Table 2:** Percent of mono- and bi-allelic expression of SNVs covered with different required minimum counts of sequencing reads. *Predominantly monoallelic expression is inclusive of strict monoallelic expression.

Sample	% Strictly Monoallelic VAF_RNA_ = 0 or 1			% Predominantly Monoallelic * VAF_RNA_ = 0–0.2 or 0.8–1			% Biallelic VAF_RNA_ = 0.2–0.8		
	minR 10	minR 5	minR 3	minR 10	minR 5	minR 3	minR 10	minR 5	minR 3
**N8**	4.4	15.1	38.2	16.1	30.6	45.5	83.9	69.4	54.5
**N7**	2.9	15.8	41.3	13.1	30.3	47.9	86.9	69.7	52.1
**N5**	2.4	12.5	35.9	10	26.1	42.0	90	76.9	58.0

## Supplementary Materials

The following are available online at https://www.mdpi.com/2073-4425/11/3/240/s1: Figure S1: Mean and median VAF_RNA_; Figure S2: VAF RNA distributions and cell counts; Table S1: SNV loci minR10 10cells; Table S2: SNV loci minR5 10cells; Table S3: SNV loci minR10 10cells.
