# Supplementary material for: Estimating the Allele-Specific Expression of SNVs From 10× Genomics Single-Cell RNA-Sequencing Data
Source: Genes (Basel). 2020 Feb 25;11(3):240. doi: 10.3390/genes11030240 (PMC7140866; doi:10.3390/genes11030240)
Supplement: Supplementary file 1 [file genes-11-00240-s001.zip › Supplementary_Figure_1_Mean_and_Median_VAFRNA.pdf]

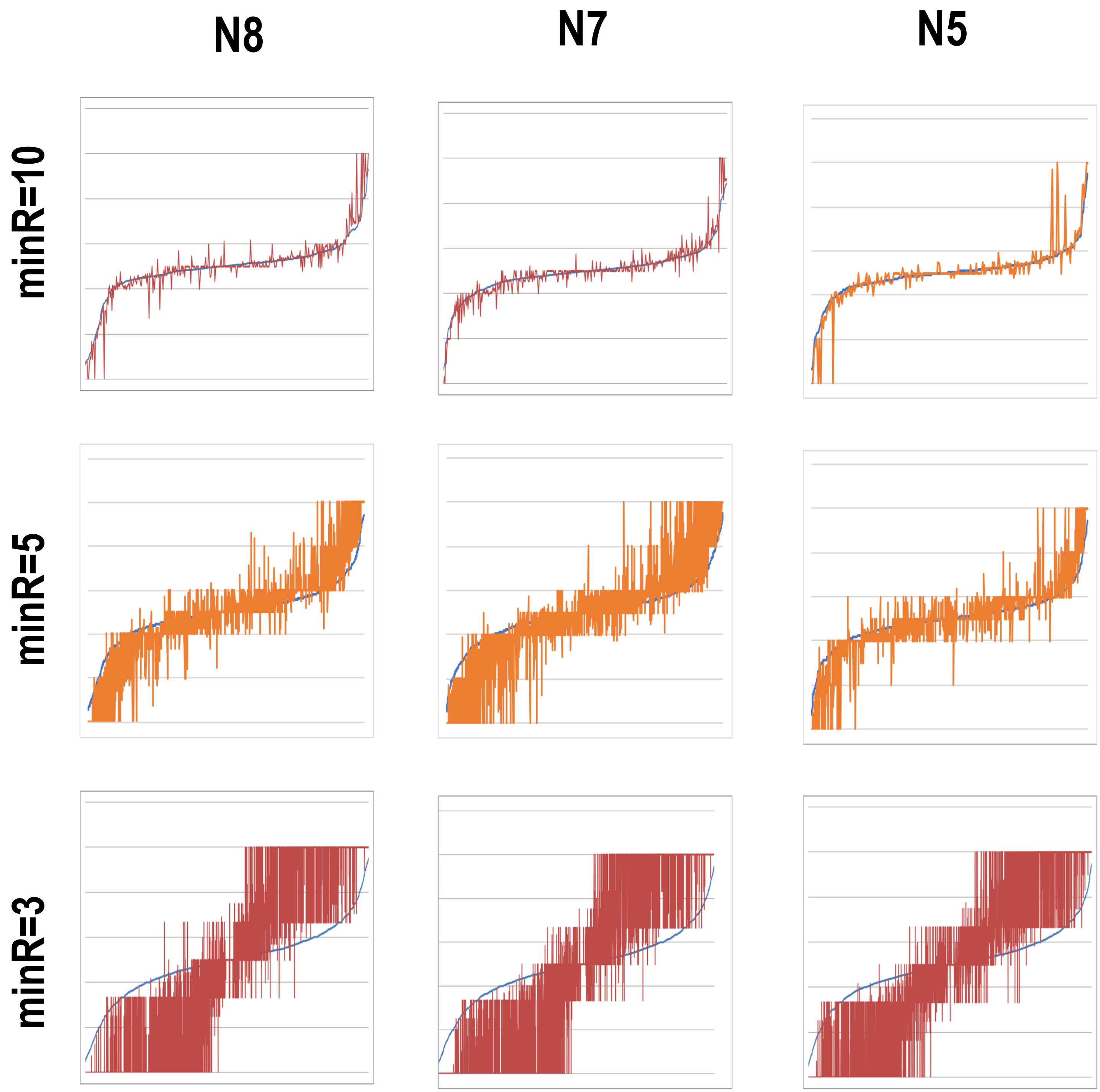

Supplementary Figure 1: Mean (blue) and Median (orange) VAF_{RNA} at different thresholds of required minimum sequencing reads (minR = 10 (top), minR = 5 (middle) and minR = 3 (bottom)/
